# On Finite Energy Solutions of 4-harmonic and ES-4-harmonic Maps

**DOI:** 10.1007/s12220-021-00610-7

**Published:** 2021-02-25

**Authors:** Volker Branding

**Affiliations:** Faculty of Mathematics, University of Vienna, Oskar-Morgenstern-Platz 1, 1090 Vienna, Austria

**Keywords:** 4-harmonic maps, ES-4-harmonic maps, Nonexistence result, Harmonic maps, 58E20, 53C43

## Abstract

4-harmonic and ES-4-harmonic maps are two generalizations of the well-studied harmonic map equation which are both given by a nonlinear elliptic partial differential equation of order eight. Due to the large number of derivatives it is very difficult to find any difference in the qualitative behavior of these two variational problems. In this article we prove that finite energy solutions of both 4-harmonic and ES-4-harmonic maps from Euclidean space must be trivial. However, the energy that we require to be finite is different for 4-harmonic and ES-4-harmonic maps pointing out a first difference between these two variational problems.

## Introduction and Results

At the heart of the geometric calculus of variations is the aim to find interesting maps between Riemannian manifolds. This can be achieved by extremizing a given energy functional.

One of the best studied energy functionals for maps between Riemannian manifolds is the energy of a map $\phi :(M,g)\rightarrow (N,h)$ which is

1.1 $$\begin{aligned} E(\phi )=\int _M|\mathrm{{d}}\phi |^2\mathrm{{d}}v. \end{aligned}$$

The critical points of (1.1) are characterized by the vanishing of the so-called *tension field* which is defined by

1.2 $$\begin{aligned} 0=\tau (\phi ):={\text {Tr}}_g\bar{\nabla }\mathrm{{d}}\phi , \end{aligned}$$

where $\bar{\nabla }$ represents the connection on $\phi ^*TN$. Solutions of (1.2) are called *harmonic maps* and the latter have been studied intensively in the literature. The harmonic map equation is a second order semilinear elliptic partial differential equation. For an overview on the current status of research on harmonic maps we refer to [9].

Recently, many researchers got attracted in energy functionals that contain higher derivatives extending the energy of a map (1.1).

A possible higher order generalization of harmonic maps is given by the so-called *polyharmonic maps of order k* or just *k-harmonic maps*. These are critical points of the following energy functionals, where we need to distinguish between polyharmonic maps of even and odd order. In the even case $(k=2s,s\in \mathbb {N})$ we set

1.3 $$\begin{aligned} E_{2s}(\phi )=\int _M|\bar{\Delta }^{s-1}\tau (\phi )|^2\mathrm{{d}}v, \end{aligned}$$

whereas in the odd case $(k=2s+1,s\in \mathbb {N})$ we have

1.4 $$\begin{aligned} E_{2s+1}(\phi )=\int _M|\bar{\nabla }\bar{\Delta }^{s-1}\tau (\phi )|^2\mathrm{{d}}v. \end{aligned}$$

Here, we use $\bar{\Delta }$ to denote the connection Laplacian on the vector bundle $\phi ^*TN$.

The first variation of (1.3), (1.4) was calculated in [12].

1. In the even case ($k=2s$), the critical points of (1.3) are given by
  1.5 $$\begin{aligned} 0&= \tau _{2s}(\phi ):=\bar{\Delta }^{2s-1}\tau (\phi )-R^N(\bar{\Delta }^{2s-2}\tau (\phi ),\mathrm{{d}}\phi (e_j))\mathrm{{d}}\phi (e_j) \nonumber \\&\quad -\,\sum _{l=1}^{s-1}\bigg (R^N(\bar{\nabla }_{e_j}\bar{\Delta }^{s+l-2}\tau (\phi ),\bar{\Delta }^{s-l-1}\tau (\phi ))\mathrm{{d}}\phi (e_j) \nonumber \\&\quad -\,R^N(\bar{\Delta }^{s+l-2}\tau (\phi ),\bar{\nabla }_{e_j}\bar{\Delta }^{s-l-1}\tau (\phi ))\mathrm{{d}}\phi (e_j) \bigg ). \end{aligned}$$
2. In the odd case ($k=2s+1$) the critical points of (1.4) are given by
  1.6 $$\begin{aligned} 0&= \tau _{2s+1}(\phi ) := \bar{\Delta }^{2s}\tau (\phi )-R^N(\bar{\Delta }^{2s-1}\tau (\phi ),\mathrm{{d}}\phi (e_j))\mathrm{{d}}\phi (e_j)\nonumber \\&\quad -\,\sum _{l=1}^{s-1}\bigg (R^N(\bar{\nabla }_{e_j}\bar{\Delta }^{s+l-1}\tau (\phi ),\bar{\Delta }^{s-l-1}\tau (\phi ))\mathrm{{d}}\phi (e_j) \nonumber \\&\quad -\,R^N(\bar{\Delta }^{s+l-1}\tau (\phi ),\bar{\nabla }_{e_j}\bar{\Delta }^{s-l-1}\tau (\phi ))\mathrm{{d}}\phi (e_j) \bigg ) \nonumber \\&\quad -\,R^N(\bar{\nabla }_{e_j}\bar{\Delta }^{s-1}\tau (\phi ),\bar{\Delta }^{s-1}\tau (\phi ))\mathrm{{d}}\phi (e_j). \end{aligned}$$

Here, we have set $\bar{\Delta }^{-1}=0$, $\{e_j\},j=1,\ldots ,m=\dim M$ denotes an orthonormal basis of $TM$, and we are applying the Einstein summation convention.

Another possible generalization of harmonic maps, first suggested by Eells and Sampson in 1964 [8], can be obtained by studying the critical points of the following energy functional

1.7 $$\begin{aligned} E^\mathrm{{ES}}_k(\phi ):=\int _M|(\mathrm{d}+\mathrm{d}^*)^k\phi |^2\mathrm{{d}}v=\int _M|(\mathrm{d}+\mathrm{d}^*)^{k-2}\tau (\phi )|^2\mathrm{{d}}v,\qquad k=1,2,\ldots \end{aligned}$$

For $k=1$ this energy functional reduces to the energy of a map (1.1).

In the case of $k=2$, which is also obtained in (1.3) for $s=1$, we are led to the bienergy $E_2(\phi )=E^\mathrm{{ES}}_2(\phi )$, whose critical points are called *biharmonic maps*. For an overview on the latter we refer to the recent book [14]. For biharmonic maps similar classification results as in this article have been obtained in [2, 4, 7].

For $k=3$ we gain the trienergy of a map $E_3(\phi )=E^\mathrm{{ES}}_3(\phi )$, which corresponds to (1.4) with $s=1$, and its critical points are called *triharmonic maps*. For an overview on triharmonic maps we refer to [5, Section 4] and references therein. Triharmonic curves have recently been studied in [13].

However, for $k\ge 4$ the energy functional (1.7) contains additional curvature terms and can in general no longer be written in the form (1.3), (1.4). An extensive analysis of (1.7) and its critical points was carried out recently in [3].

This article is devoted to polyharmonic maps of order $4$ arising either as critical points of (1.3) or (1.7).

The energy functional for 4-harmonic maps (corresponding to (1.3) with $s=2$) is given by

1.8 $$\begin{aligned} E_4(\phi )=\int _M|\bar{\Delta }\tau (\phi )|^2\mathrm{{d}}v. \end{aligned}$$

The critical points of (1.8) are characterized by the vanishing of the 4-tension field

1.9 $$\begin{aligned} 0&= \tau _{4}(\phi ) := \bar{\Delta }^{3}\tau (\phi )-R^N(\bar{\Delta }^{2}\tau (\phi ),\mathrm{{d}}\phi (e_j))\mathrm{{d}}\phi (e_j)\nonumber \\&\quad +\,R^N(\tau (\phi ),\bar{\nabla }_{e_j}\bar{\Delta }\tau (\phi ))\mathrm{{d}}\phi (e_j) -R^N(\bar{\nabla }_{e_j}\tau (\phi ),\bar{\Delta }\tau (\phi ))\mathrm{{d}}\phi (e_j). \end{aligned}$$

Solutions of (1.9) are called *4-harmonic maps*.

The energy functional for ES-4-harmonic maps (corresponding to (1.7) with $k=4$) is given by

1.10 $$\begin{aligned} E^\mathrm{{ES}}_4(\phi )&=\int _M|(\mathrm{d}+\mathrm{d}^*)^4\phi |^2\mathrm{{d}}v\nonumber \\&=\int _M|\bar{\Delta }\tau (\phi )|^2\mathrm{{d}}v +\frac{1}{2}\int _M|R^N(\mathrm{{d}}\phi (e_i),\mathrm{{d}}\phi (e_j))\tau (\phi )|^2\mathrm{{d}}v. \end{aligned}$$

The first variation of (1.10) was calculated in [3, Section 3] and is characterized by the vanishing of the ES-4-tension field $\tau _4^\mathrm{{ES}}(\varphi )$ given by the following expression

1.11 $$\begin{aligned} {\tau _{4}}^\mathrm{{ES}}(\phi )={\tau _{4}}(\phi )+{\hat{\tau }_{4}}(\phi ). \end{aligned}$$

Here, $\tau _4(\phi )$ denotes the 4-tension field (1.9) and the quantity ${\hat{\tau }_{4}}(\phi )$ is defined by

$$\begin{aligned} {\hat{\tau }_{4}}(\phi )=-\frac{1}{2}\big (2\xi _1+2\mathrm{{d}}^*\Omega _1+\bar{\Delta }\Omega _0+{\text {Tr}}R^N(\mathrm{{d}}\phi (\cdot ),\Omega _0)\mathrm{{d}}\phi (\cdot )\big ), \end{aligned}$$

where we have used the following abbreviations

1.12 $$\begin{aligned} \Omega _0&=R^N(\mathrm{{d}}\phi (e_i),\mathrm{{d}}\phi (e_j))R^N(\mathrm{{d}}\phi (e_i),\mathrm{{d}}\phi (e_j))\tau (\phi ), \nonumber \\ \Omega _1(X)&=R^N(R^N(\mathrm{{d}}\phi (X),\mathrm{{d}}\phi (e_j))\tau (\phi ),\tau (\phi ))\mathrm{{d}}\phi (e_j),\nonumber \\ \xi _1&=-(\nabla _{\mathrm{{d}}\phi (e_j)}R^N)(R^N(\mathrm{{d}}\phi (e_i),\mathrm{{d}}\phi (e_j))\tau (\phi ),\tau (\phi ))\mathrm{{d}}\phi (e_i). \end{aligned}$$

Note that we use a slightly different notation for the $\xi _1$ term in (1.12) compared to [3].

It can be directly seen that both constant and harmonic maps are absolute minimizers of the higher order energy functionals (1.3), (1.4) and (1.7). In order to understand the mathematical structure of these energy functionals it seems important to find conditions that force critical points of these functionals to be constant or harmonic maps.

We will prove the following results for finite energy solutions of (1.9)

### Theorem 1.1

Let $\phi :\mathbb {R}^m\rightarrow N$ be a smooth 4-harmonic map, $m\ne 8$. Assume that

1.13 $$\begin{aligned} \int _{\mathbb {R}^m}(|\mathrm{{d}}\phi |^2+|\bar{\nabla }\mathrm{{d}}\phi |^2+|\bar{\nabla }^2\mathrm{{d}}\phi |^2+|\bar{\nabla }^3\mathrm{{d}}\phi |^2)\mathrm{{d}}v<\infty . \end{aligned}$$

If $m=2$ then $\phi$ must be harmonic, if $m>2$ then $\phi$ must be constant.

The second main result of this article is the following result on finite energy solutions of (1.11)

### Theorem 1.2

Let $\phi :\mathbb {R}^m\rightarrow N$ be a smooth ES-4-harmonic map, $m\ne 8$ and suppose that $|R^N|_{L^\infty }<\infty$. Assume that

1.14 $$\begin{aligned} \int _{\mathbb {R}^m}(|\mathrm{{d}}\phi |^2+|\bar{\nabla }\mathrm{{d}}\phi |^2+|\bar{\nabla }^2\mathrm{{d}}\phi |^2+|\bar{\nabla }^3\mathrm{{d}}\phi |^2+|\mathrm{{d}}\phi |^4|\bar{\nabla }\mathrm{{d}}\phi |^2+|\mathrm{{d}}\phi |^6)\mathrm{{d}}v<\infty . \end{aligned}$$

If $m=2$ then $\phi$ must be harmonic, if $m>2$ then $\phi$ must be constant.

### Remark 1.3

1. Due to the last two terms in (1.14) the assumptions of Theorem 1.2 are more restrictive than the assumptions of Theorem 1.1. This points out a first difference between 4-harmonic and ES-4-harmonic maps.
2. It should be stressed that the assumptions (1.13), (1.14) are stronger than demanding the finiteness of the 4-energy (1.8) or the ES-4-energy (1.10). A similar phenomenon also appears in corresponding results for biharmonic [2, Theorem 3.4] and triharmonic [5, Theorem 4.1] maps.
3. The terminology “finite energy solutions” is usually employed in corresponding results for harmonic maps with finite energy (1.1). In this paper, we use the same wording to denote solutions of (1.9) satisfying (1.13) and also solutions of (1.11) satisfying (1.14).

In addition to Theorems 1.1 and 1.2 we want to mention another result characterizing the behavior of 4-harmonic maps which is a special case of a structure theorem for polyharmonic maps established in [6]. As the proof of this theorem relies on a different method we have to make the additional assumption that $N$ has bounded geometry which means that its curvature tensor and all of its covariant derivatives are bounded.

### Theorem 1.4

Let $\phi :\mathbb {R}^m\rightarrow N$ be a smooth 4-harmonic map, $m>6$ and suppose that $N$ has bounded geometry.

1. Suppose that the following condition holds
  $$\begin{aligned} \int _{\mathbb {R}^m}(|\mathrm{{d}}\phi |^m+|\bar{\nabla }\mathrm{{d}}\phi |^\frac{m}{2}+|\bar{\nabla }^2\mathrm{{d}}\phi |^\frac{m}{3})\mathrm{{d}}v<\varepsilon \end{aligned}$$
  for some $\varepsilon >0$ small enough.
2. In addition, assume that
  $$\begin{aligned} \int _{\mathbb {R}^m}(|\bar{\Delta }\tau (\phi )|^2+|\bar{\nabla }\bar{\Delta }\tau (\phi )|^2+|\bar{\nabla }^2\bar{\Delta }\tau (\phi )|^2)\mathrm{{d}}v<\infty . \end{aligned}$$

Then $\phi$ must be harmonic.

This result can also be extended to the case of ES-4-harmonic maps. At the heart of the proof of Theorem 1.4 is a Sobolev inequality which is used to control the lower order terms on the right hand side of (1.9). However, the extension of this technique to ES-4-harmonic maps would require that $m>10$ and also $\tau (\phi )\in W^{4,2}(\mathbb {R}^m,\phi ^*TN)$ in addition to the assumptions made in Theorem 1.2.

We would like to point out that the method of proof used for Theorems 1.1 and 1.2 only seems to work on Euclidean space as we are making use of a globally defined conformal vector field. On the other hand, the method of proof used for Theorem 1.4 is not restricted to $\mathbb {R}^m$ but works on all manifolds that admit a *Euclidean type Sobolev inequality*. For more details on the latter see the introduction of [6] and references therein.

Theorems 1.1 and 1.2 make use of the stress-energy tensor. For harmonic maps this tensor was calculated in [1], for biharmonic maps it was given in [10] and later systematically derived in [11]. For polyharmonic maps the stress-energy tensor was obtained recently in [5].

Throughout this article, we will use the following notation: Indices on the domain manifold will be denoted by Latin letters $i=1,\ldots ,m=\dim M$ and we will employ Greek letters $\alpha =1,\ldots ,n=\dim N$ for indices on the target manifold. We will use the following sign convention for the *rough Laplacian* acting on sections of $\phi ^{*}TN$

$$\begin{aligned} {\bar{\Delta }}=\mathrm{d}^*\mathrm{d} =-\big (\bar{\nabla }_{e_i}\bar{\nabla }_{e_i}-\bar{\nabla }_{\nabla ^M_{e_i}e_i}\big ), \end{aligned}$$

where $\{e_i\},i=1,\ldots m$ is a local orthonormal frame field tangent to *M*. Moreover, we employ the summation convention and tacitly sum over repeated indices. We will often write $\bar{\nabla }_i$ instead of $\bar{\nabla }_{e_i}$. Throughout this article we make use of the following sign convention for the curvature of a connection

$$\begin{aligned} R(X,Y)Z=\nabla _X\nabla _YZ-\nabla _Y\nabla _XZ-\nabla _{[X,Y]}Z \end{aligned}$$

for given vector fields $X,Y,Z$. The letter $C$ will always represent a positive constant whose value may change from line to line.

This article is organized as follows: In Sect. 2 we prove Theorem 1.1. Afterwards, in Sect. 3, we derive the stress-energy tensor for ES-4-harmonic maps and employ it in Sect. 4 to prove Theorem 1.2.

## Proof of Theorem 1.1

In this section, we will prove Theorem 1.1. The proof relies on the stress energy-tensor associated with (1.8) which can be obtained by varying (1.8) with respect to the metric on the domain. This variation was carried out in detail in [5, Section 2], the resulting stress-energy tensor is given by

2.1 $$\begin{aligned} S_{4}(X,Y)&:=g(X,Y)\bigg (-\frac{1}{2}|\bar{\Delta }\tau (\phi )|^2-\langle \tau (\phi ),\bar{\Delta }^{2}\tau (\phi )\rangle -\langle \mathrm{{d}}\phi ,\bar{\nabla }\bar{\Delta }^{2}\tau (\phi )\rangle \nonumber \\&\quad +\,\langle \bar{\nabla }\tau (\phi ),\bar{\nabla }\bar{\Delta }\tau (\phi )\rangle \bigg ) \nonumber \\&\quad -\,\langle \bar{\nabla }_X\tau (\phi ),\bar{\nabla }_Y\bar{\Delta }\tau (\phi )\rangle -\langle \bar{\nabla }_Y\bar{\Delta }\tau (\phi ),\bar{\nabla }_X\bar{\Delta }\tau (\phi )\rangle \nonumber \\&\quad +\,\langle \mathrm{{d}}\phi (X),\bar{\nabla }_Y\bar{\Delta }^{2}\tau (\phi )\rangle +\langle \mathrm{{d}}\phi (Y),\bar{\nabla }_X\bar{\Delta }^{2}\tau (\phi )\rangle . \end{aligned}$$

It was also shown in [5, Proposition 2.6] that the stress-energy tensor (2.1) satisfies the following conservation law:

### Proposition 2.1

Let $\phi :M\rightarrow N$ be a smooth map. Then the stress-energy tensor (2.1) satisfies the following conservation law

2.2 $$\begin{aligned} {\text {div}}S_{4}=-\langle \tau _{4}(\phi ),\mathrm{{d}}\phi \rangle . \end{aligned}$$

In particular, $S_4$ is divergence-free whenever $\phi$ is a 4-harmonic map, that is a solution of (1.9).

Now, for $R>0$ let $\eta \in C_0^\infty (\mathbb {R})$ be a smooth cut-off function satisfying $\eta =1$ for $|z|\le R$, $\eta =0$ for $|z|\ge 2R$ and $|\eta ^l(z)|\le \frac{C}{R^l},l=1,\ldots ,4$. We define the function $Y(x):=x\eta (r)\in C_0^\infty (\mathbb {R}^m,\mathbb {R}^m)$ with $r=|x|$. It follows directly that

$$\begin{aligned} \frac{\partial Y_i}{\partial x^j}=\delta _{ij}\eta (r)+\frac{x_i x_j}{r}\eta '(r). \end{aligned}$$

Due to the conservation law (2.2) we have

$$\begin{aligned} 0=-\int _{\mathbb {R}^m}\langle Y,{\text {div}}S_4\rangle \mathrm{{d}}v=\int _{\mathbb {R}^m}\frac{\partial Y_i}{\partial x^j}S_4(e_i,e_j)\mathrm{{d}}v. \end{aligned}$$

By a direct computation we find

2.3 $$\begin{aligned} \int _{\mathbb {R}^m} S_4(e_i,e_j)\delta _{ij}\eta (r)\mathrm{{d}}v&= \int _{\mathbb {R}^m}\eta (r)\big (-\,m(\frac{1}{2}|\bar{\Delta }\tau (\phi )|^2\nonumber \\&\quad +\,\langle \tau (\phi ),\bar{\Delta }^2\tau (\phi )\rangle ) + \,(2-m)\langle \mathrm{{d}}\phi ,\bar{\nabla }\bar{\Delta }^2\tau (\phi )\rangle \nonumber \\&\quad +\,(m-2)\langle \bar{\nabla }\tau (\phi ),\bar{\nabla }\bar{\Delta }\tau (\phi )\rangle \big )\mathrm{{d}}v\nonumber \\&:= \sum _{r=1}^4H_r. \end{aligned}$$

As a next step we manipulate the four terms on the right hand side of (2.3). Note that the $H_1$-term already has the form that we need. Hence, we start by manipulating the $H_2$-term as follows

$$\begin{aligned} \int _{\mathbb {R}^m}\eta (r)\langle \tau (\phi ),\bar{\Delta }^2\tau (\phi )\rangle \mathrm{{d}}v&= -\,\int _{\mathbb {R}^m}\big (\eta (r)\big )_{jj}\langle \tau (\phi ),\bar{\Delta }\tau (\phi )\rangle \mathrm{{d}}v \\&\quad -\,2\int _{\mathbb {R}^m}\big (\eta (r)\big )_{j}\langle \bar{\nabla }_j\tau (\phi ),\bar{\Delta }\tau (\phi )\rangle \mathrm{{d}}v\\&\quad +\,\int _{\mathbb {R}^m}\eta (r)|\bar{\Delta }\tau (\phi )|^2\mathrm{{d}}v. \end{aligned}$$

Here, and in the following, a subscript $j$ denotes the derivative with respect to the $j$-th coordinate variable in $\mathbb {R}^m$.

For the $H_3$-term we obtain

$$\begin{aligned} \int _{\mathbb {R}^m}\eta (r)\langle \mathrm{{d}}\phi ,\bar{\nabla }\bar{\Delta }^2\tau (\phi )\rangle \mathrm{{d}}v&= -\,\int _{\mathbb {R}^m}\eta (r)\langle \tau (\phi ),\bar{\Delta }^2\tau (\phi )\rangle \mathrm{{d}}v\\&\quad +\,\int _{\mathbb {R}^m}\big (\eta (r)\big )_{jkk}\langle \mathrm{{d}}\phi (e_j),\bar{\Delta }\tau (\phi )\rangle \mathrm{{d}}v \\&\quad +\,2\int _{\mathbb {R}^m}\big (\eta (r)\big )_{jk}\langle \bar{\nabla }_k \mathrm{{d}}\phi (e_j),\bar{\Delta }\tau (\phi )\rangle \mathrm{{d}}v \\&\quad -\,\int _{\mathbb {R}^m}\big (\eta (r)\big )_j\langle \bar{\Delta }\mathrm{{d}}\phi (e_j),\bar{\Delta }\tau (\phi )\rangle \mathrm{{d}}v. \end{aligned}$$

Regarding the $H_4$-term we get

$$\begin{aligned} \int _{\mathbb {R}^m}\eta (r)\langle \bar{\nabla }\tau (\phi ),\bar{\nabla }\bar{\Delta }\tau (\phi )\rangle \mathrm{{d}}v&= \int _{\mathbb {R}^m}\eta (r)|\bar{\Delta }\tau (\phi )|^2\mathrm{{d}}v\\&\quad -\,\int _{\mathbb {R}^m}\big (\eta (r)\big )_j\langle \bar{\nabla }_j\tau (\phi ),\bar{\Delta }\tau (\phi )\rangle \mathrm{{d}}v. \end{aligned}$$

By another direct computation we obtain

2.4 $$\begin{aligned} \int _{\mathbb {R}^m} S_4(e_i,e_j)\frac{x_i x_j}{r}\eta '(r)\mathrm{{d}}v&= \int _{\mathbb {R}^m}\eta '(r)r\left( -\frac{1}{2}|\bar{\Delta }\tau (\phi )|^2-\langle \tau (\phi ),\bar{\Delta }^2\tau (\phi )\rangle \right) \nonumber \\&\quad -\,\langle \mathrm{{d}}\phi ,\bar{\nabla }\bar{\Delta }^2\tau (\phi )\rangle +\langle \bar{\nabla }\tau (\phi ),\bar{\nabla }\bar{\Delta }\tau (\phi )\rangle \big )\mathrm{{d}}v \nonumber \\&\quad +\,2\int _{\mathbb {R}^m}\eta '(r)\frac{x_i x_j}{r}\langle \mathrm{{d}}\phi (e_i),\bar{\nabla }_j\bar{\Delta }^2\tau (\phi )\rangle \mathrm{{d}}v \nonumber \\&\quad -\,2\int _{\mathbb {R}^m}\eta '(r)\frac{x_i x_j}{r}\langle \bar{\nabla }_i\tau (\phi ),\bar{\nabla }_j\bar{\Delta }\tau (\phi )\rangle \mathrm{{d}}v\nonumber \\&:= \sum _{r=1}^6J_r. \end{aligned}$$

Similar as before, we will now manipulate the $J_r$-terms, $r=2,\ldots ,6$ using integration by parts, the $J_1$-term already has the desired form. Regarding the $J_2$ and the $J_3$-terms we find

$$\begin{aligned} J_3&= -J_2-\int _{\mathbb {R}^m}\big (r\eta '(r)\big )_{jkk}\langle \mathrm{{d}}\phi (e_j),\bar{\Delta }\tau (\phi )\rangle \mathrm{{d}}v\\&\quad -\,2\int _{\mathbb {R}^m}\big (r\eta '(r)\big )_{jk}\langle \bar{\nabla }_k \mathrm{{d}}\phi (e_j),\bar{\Delta }\tau (\phi )\rangle \mathrm{{d}}v \\&\quad +\,\int _{\mathbb {R}^m}\big (r\eta '(r)\big )_{j}\langle \bar{\Delta }\mathrm{{d}}\phi (e_j),\bar{\Delta }\tau (\phi )\rangle \mathrm{{d}}v. \end{aligned}$$

The $J_4$-term can easily manipulated to give

$$\begin{aligned} J_4=\int _{\mathbb {R}^m}\eta '(r)r|\bar{\Delta }\tau (\phi )|^2\mathrm{{d}}v -\int _{\mathbb {R}^m}\big (r\eta '(r)\big )_j\langle \bar{\nabla }_j\tau (\phi ),\bar{\Delta }\tau (\phi )\rangle \mathrm{{d}}v. \end{aligned}$$

Using integration by parts several times we can express the $J_5$-term as

$$\begin{aligned} \frac{J_5}{2}&=\int _{\mathbb {R}^m}\left( \eta '(r)\frac{x_i x_j}{r}\right) _{jkk}\langle \mathrm{{d}}\phi (e_i),\bar{\Delta }\tau (\phi )\rangle \mathrm{{d}}v\\&\quad +2\int _{\mathbb {R}^m}\left( \eta '(r)\frac{x_i x_j}{r}\right) _{jk}\langle \bar{\nabla }_k \mathrm{{d}}\phi (e_i),\bar{\Delta }\tau (\phi )\rangle \mathrm{{d}}v \\&\quad -\int _{\mathbb {R}^m}\left( \eta '(r)\frac{x_i x_j}{r}\right) _{j}\langle \bar{\Delta }\mathrm{{d}}\phi (e_i),\bar{\Delta }\tau (\phi )\rangle \mathrm{{d}}v\\&\quad +\int _{\mathbb {R}^m}\left( \eta '(r)\frac{x_i x_j}{r}\right) _{kk}\langle \bar{\nabla }_j \mathrm{{d}}\phi (e_i),\bar{\Delta }\tau (\phi )\rangle \mathrm{{d}}v \\&\quad +2\int _{\mathbb {R}^m}\left( \eta '(r)\frac{x_i x_j}{r}\right) _{k}\langle \bar{\nabla }_k\bar{\nabla }_j \mathrm{{d}}\phi (e_i),\bar{\Delta }\tau (\phi )\rangle \mathrm{{d}}v\\&\quad -\int _{\mathbb {R}^m}\eta '(r)\frac{x_i x_j}{r}\langle \bar{\Delta }\bar{\nabla }_j \mathrm{{d}}\phi (e_i),\bar{\Delta }\tau (\phi )\rangle \mathrm{{d}}v. \end{aligned}$$

Finally, for the $J_6$-term we get

$$\begin{aligned} \frac{J_6}{2}&= \int _{\mathbb {R}^m}\eta '(r)\frac{x_i x_j}{r}\langle \bar{\nabla }_j\bar{\nabla }_i\tau (\phi ),\bar{\Delta }\tau (\phi )\rangle \mathrm{{d}}v\\&\quad +\,\int _{\mathbb {R}^m}\left( \eta '(r)\frac{x_ix_j}{r}\right) _j\langle \bar{\nabla }_i\tau (\phi ),\bar{\Delta }\tau (\phi )\rangle \mathrm{{d}}v. \end{aligned}$$

Combining (2.3) and (2.4) and using the identities for $H_r,r=1,\ldots 4$ and $J_r,r=1\ldots 6$ we can deduce that

2.5 $$\begin{aligned}&\left( 4-\frac{m}{2}\right) \int _{\mathbb {R}^m}\eta (r)|\bar{\Delta }\tau (\phi )|^2\mathrm{{d}}v\nonumber \\&\quad = 2\int _{\mathbb {R}^m}\big (\eta (r)\big )_{jj}\langle \tau (\phi ),\bar{\Delta }\tau (\phi )\rangle \mathrm{{d}}v -(m-6)\int _{\mathbb {R}^m}\big (\eta (r)\big )_{j}\langle \bar{\nabla }_j\tau (\phi ),\bar{\Delta }\tau (\phi )\rangle \mathrm{{d}}v \nonumber \\&\quad \quad +\,(2-m)\int _{\mathbb {R}^m}\big (\eta (r)\big )_{jkk}\langle \mathrm{{d}}\phi (e_j),\bar{\Delta }\tau (\phi )\rangle \mathrm{{d}}v \nonumber \\&\quad \quad -\,(2-m)\int _{\mathbb {R}^m}\big (\eta (r)\big )_j\langle \bar{\Delta }\mathrm{{d}}\phi (e_j),\bar{\Delta }\tau (\phi )\rangle \mathrm{{d}}v\nonumber \\&\quad \quad +\,2(2-m)\int _{\mathbb {R}^m}\big (\eta (r)\big )_{jk}\langle \bar{\nabla }_k \mathrm{{d}}\phi (e_j),\bar{\Delta }\tau (\phi )\rangle \mathrm{{d}}v \nonumber \\&\quad \quad +\,\frac{1}{2}\int _{\mathbb {R}^m}\eta '(r)r|\bar{\Delta }\tau (\phi )|^2\mathrm{{d}}v -\int _{\mathbb {R}^m}\big (r\eta '(r)\big )_j\langle \bar{\nabla }_j\tau (\phi ),\bar{\Delta }\tau (\phi )\rangle \mathrm{{d}}v \nonumber \\&\quad \quad -\,\int _{\mathbb {R}^m}\big (r\eta '(r)\big )_{jkk}\langle \mathrm{{d}}\phi (e_j),\bar{\Delta }\tau (\phi )\rangle \mathrm{{d}}v \nonumber \\&\qquad -\,2\int _{\mathbb {R}^m}\big (r\eta '(r)\big )_{jk}\langle \bar{\nabla }_k \mathrm{{d}}\phi (e_j),\bar{\Delta }\tau (\phi )\rangle \mathrm{{d}}v \nonumber \\&\qquad +\,\int _{\mathbb {R}^m}\big (r\eta '(r)\big )_{j}\langle \bar{\Delta }\mathrm{{d}}\phi (e_j),\bar{\Delta }\tau (\phi )\rangle \mathrm{{d}}v \nonumber \\&\qquad +\,2\int _{\mathbb {R}^m}\left( \eta '(r)\frac{x_i x_j}{r}\right) _{jkk}\langle \mathrm{{d}}\phi (e_i),\bar{\Delta }\tau (\phi )\rangle \mathrm{{d}}v\nonumber \\&\qquad +\,4\int _{\mathbb {R}^m}\left( \eta '(r)\frac{x_i x_j}{r}\right) _{jk}\langle \bar{\nabla }_k \mathrm{{d}}\phi (e_i),\bar{\Delta }\tau (\phi )\rangle \mathrm{{d}}v \nonumber \\&\qquad -\,2\int _{\mathbb {R}^m}\left( \eta '(r)\frac{x_i x_j}{r}\right) _{j}\langle \bar{\Delta }\mathrm{{d}}\phi (e_i),\bar{\Delta }\tau (\phi )\rangle \mathrm{{d}}v \nonumber \\&\qquad +\,2\int _{\mathbb {R}^m}\left( \eta '(r)\frac{x_i x_j}{r}\right) _{kk}\langle \bar{\nabla }_j \mathrm{{d}}\phi (e_i),\bar{\Delta }\tau (\phi )\rangle \mathrm{{d}}v \nonumber \\&\qquad +\,4\int _{\mathbb {R}^m}\left( \eta '(r)\frac{x_i x_j}{r}\right) _{k}\langle \bar{\nabla }_k\bar{\nabla }_j \mathrm{{d}}\phi (e_i),\bar{\Delta }\tau (\phi )\rangle \mathrm{{d}}v\nonumber \\&\qquad -\,2\int _{\mathbb {R}^m}\eta '(r)\frac{x_i x_j}{r}\langle \bar{\Delta }\bar{\nabla }_j \mathrm{{d}}\phi (e_i),\bar{\Delta }\tau (\phi )\rangle \mathrm{{d}}v \nonumber \\&\qquad +\,2\int _{\mathbb {R}^m}\eta '(r)\frac{x_i x_j}{r}\langle \bar{\nabla }_j\bar{\nabla }_i\tau (\phi ),\bar{\Delta }\tau (\phi )\rangle \mathrm{{d}}v\nonumber \\&\qquad +\,2\int _{\mathbb {R}^m}\big (\eta '(r)\frac{x_ix_j}{r}\big )_j\langle \bar{\nabla }_i\tau (\phi ),\bar{\Delta }\tau (\phi )\rangle \mathrm{{d}}v. \end{aligned}$$

In order to estimate the terms on the right hand side of (2.5) we perform the following direct calculations and estimate

2.6 $$\begin{aligned} \big (\eta (r)\big )_{jkk}&=\eta '''(r)\frac{x_j}{r}\nonumber \\&\le \frac{C}{R^3},\nonumber \\ \big (\eta (r)\big )_{jk}&=\eta ''(r)\frac{x_kx_j}{r^2}+\eta '(r)\frac{\delta _{jk}}{r}-\eta '(r)\frac{x_jx_k}{r^3}\nonumber \\&\le \frac{C}{R^2},\nonumber \\ \big (\eta (r)\big )_{j}&=\eta '(r)\frac{x_j}{r}\nonumber \\&\le \frac{C}{R}. \end{aligned}$$

Similarly, we obtain

2.7 $$\begin{aligned} \left( \eta '(r)\frac{x_i x_j}{r}\right) _{jkk}&=\eta ^{(4)}(r)x_i+3\eta '''(r)\frac{x_i}{r} \nonumber \\&\le \frac{C}{R^3},\nonumber \\ \left( \eta '(r)\frac{x_i x_j}{r}\right) _{jk}&=\eta '''(r)\frac{x_ix_k}{r}+\eta ''(r)\delta _{ik} +\eta ''(r)\frac{x_ix_k}{r^2}+\eta '(r)\frac{\delta _{ik}}{r}-\eta '(r)\frac{x_ix_k}{r^3}\nonumber \\&\le \frac{C}{R^2},\nonumber \\ \left( \eta '(r)\frac{x_i x_j}{r}\right) _{kk}&=\eta '''(r)\frac{x_ix_j}{r} +2\eta ''(r)\frac{x_ix_j}{r^2}+2\eta '(r)\frac{\delta _{ij}}{r}-2\eta '(r)\frac{x_ix_j}{r^3}\nonumber \\&\le \frac{C}{R^2},\nonumber \\ \left( \eta '(r)\frac{x_i x_j}{r}\right) _{k}&=\eta ''(r)\frac{x_ix_jx_k}{r^2}+\eta '(r)\frac{\delta _{ik}x_j}{r} +\eta '(r)\frac{\delta _{jk}x_i}{r} -\eta '(r)\frac{x_ix_jx_k}{r^3}\nonumber \\&\le \frac{C}{R}. \end{aligned}$$

Inserting (2.6) and (2.7) into (2.5) and using Young’s inequality multiple times we find

$$\begin{aligned} \int _{\mathbb {R}^m}\eta (r)|\bar{\Delta }\tau (\phi )|^2\mathrm{{d}}v&\le \frac{C}{|8-m|}\left( \frac{1}{R}+\frac{1}{R^2}+\frac{1}{R^3}\right) \\&\quad \times \,\int _{\mathbb {R}^m}(|\mathrm{{d}}\phi |^2+|\bar{\nabla }\mathrm{{d}}\phi |^2+|\bar{\nabla }^2\mathrm{{d}}\phi |^2+|\bar{\nabla }^3\mathrm{{d}}\phi |^2)\mathrm{{d}}v \\&\quad +\,\frac{C}{|8-m|}\int _{B_{2R}\setminus B_R}|\bar{\Delta }\tau (\phi )|^2\mathrm{{d}}v. \end{aligned}$$

Taking the limit $R\rightarrow \infty$ and using the finiteness assumption (1.13) the calculation from above yields that $\bar{\Delta }\tau (\phi )=0$.

At this point, we employ integration by parts

$$\begin{aligned} 0&= -\,\int _{\mathbb {R}^m}\eta ^2\langle \underbrace{\bar{\Delta }\tau (\phi )}_{=0},\tau (\phi )\rangle \mathrm{{d}}v=-\int _{\mathbb {R}^m}\eta ^2|\bar{\nabla }\tau (\phi )|^2\mathrm{{d}}v\\&\quad -\,2\int _{\mathbb {R}^m}\eta \nabla \eta \langle \bar{\nabla }\tau (\phi ),\tau (\phi )\rangle \mathrm{{d}}v \end{aligned}$$

from which we may deduce that

$$\begin{aligned} \int _{\mathbb {R}^m}\eta ^2|\bar{\nabla }\tau (\phi )|^2\mathrm{{d}}v\le \frac{C}{R^2}\int _{\mathbb {R}^m}|\tau (\phi )|^2\mathrm{{d}}v\le \frac{C}{R^2}\int _{\mathbb {R}^m}|\bar{\nabla }\mathrm{{d}}\phi |^2\mathrm{{d}}v. \end{aligned}$$

Again, taking the limit $R\rightarrow \infty$ yields that $\bar{\nabla }\tau (\phi )=0$. Testing $\bar{\nabla }\tau (\phi )=0$ with $-\eta ^2\mathrm{{d}}\phi$ and performing the same step as before we can conclude that $\tau (\phi )=0$.

Now, the claim that $\phi$ must be trivial if $m\ne 2$ follows from a classical result of Sealey [15, Corollary 1] which states that there does not exist a non-constant harmonic map of finite energy $E(\phi )=\int _{\mathbb {R}^m}|\mathrm{{d}}\phi |^2\mathrm{{d}}v$ if the domain is the Euclidean space $\mathbb {R}^m,m\ge 3$ with the flat metric.

## The Stress-Energy Tensor for ES-4-harmonic Maps

In this section, we derive the stress-energy tensor associated with ES-4-harmonic maps by varying the functional $E^\mathrm{{ES}}_4(\phi )$ with respect to the metric on the domain. We only have to compute the variation with respect to the metric of the second term in (1.10) as the stress-energy tensor for 4-harmonic maps was already derived in [5, Section 2]. In this section we will allow $(M,g)$ to be an arbitrary Riemannian manifold and do not restrict to the case $M=\mathbb {R}^m$.

Throughout this section we set

3.1 $$\begin{aligned} \frac{\text {d}}{\text {d}t}\big |_{t=0}g_{ij}=\omega _{ij}, \end{aligned}$$

where $\omega _{ij}$ is a smooth symmetric $2$-tensor on $M$.

### Lemma 3.1

Let $\phi :M\rightarrow N$ be a smooth map and consider a variation of the metric on $M$ as defined in (3.1). Then the following formula holds

3.2 $$\begin{aligned} \frac{\mathrm {d}}{\mathrm {d}t}\big |_{t=0}\frac{1}{2}\int _M&|R^N({\mathrm {d}}\phi (e_k),\mathrm{{d}}\phi (e_l))\tau (\phi )|^2\mathrm{{d}}v_{g_t} \nonumber \\&= \int _M\langle R^N(\mathrm{{d}}\phi (e_k),\mathrm{{d}}\phi (e_l))\frac{\mathrm {d}}{\mathrm {d}t}\big |_{t=0}\tau (\phi ),R^N(\mathrm{{d}}\phi (e_k),\mathrm{{d}}\phi (e_l))\tau (\phi )\rangle \mathrm{{d}}v_{g} \nonumber \\&\quad -\,\int _M\langle R^N(\mathrm{{d}}\phi (e_k),\mathrm{{d}}\phi (e_i))\tau (\phi ),R^N(\mathrm{{d}}\phi (e_k),\mathrm{{d}}\phi (e_j))\tau (\phi )\rangle \omega ^{ij}\mathrm{{d}}v_{g}\nonumber \\&\quad +\,\frac{1}{4}\int _M|R^N(\mathrm{{d}}\phi (e_k),\mathrm{{d}}\phi (e_l))\tau (\phi )|^2\langle \omega ,g\rangle \mathrm{{d}}v_{g}. \end{aligned}$$

### Proof

Recall that the variation of the volume element is given by

$$\begin{aligned} \frac{\text {d}}{\text {d}t}|_{t=0}\mathrm{{d}}v_{g_t}=\frac{1}{2}\langle g,\omega \rangle \mathrm{{d}}v_g. \end{aligned}$$

The claim then follows by a direct calculation using that

$$\begin{aligned} \frac{\mathrm {d}}{\mathrm {d}t}\big |_{t=0}g^{ij}=-\omega ^{ij}. \end{aligned}$$

$$\square $$

To proceed we recall the following lemma (see for example [5, Lemma 2.2])

### Lemma 3.2

Let $\phi :M\rightarrow N$ be a smooth map and consider a variation of the metric on $M$ as defined in (3.1). The variation of the tension field with respect to the metric on the domain is given by

3.3 $$\begin{aligned} \frac{\mathrm {d}}{\mathrm {d}t}\big |_{t=0}\tau ^\alpha (\phi )=-\omega ^{ij}(\bar{\nabla }\mathrm{{d}}\phi )^\alpha _{ij}-(\nabla _i\omega ^{ki})\mathrm{{d}}\phi ^\alpha (e_k) +\frac{1}{2}(\nabla ^k{\text {Tr}}\omega )\mathrm{{d}}\phi ^\alpha (e_k), \end{aligned}$$

where $$\alpha =1,\ldots ,n$$.

This allows us to perform the following computation:

### Lemma 3.3

Let $\phi :M\rightarrow N$ be a smooth map and consider a variation of the metric on $M$ as defined in (3.1). Then, the following formula holds

3.4 $$\begin{aligned} \int _M\langle&R^N(\mathrm{{d}}\phi (e_k),\mathrm{{d}}\phi (e_l))\frac{\mathrm {d}}{\mathrm {d}t}\big |_{t=0}\tau (\phi ),R^N(\mathrm{{d}}\phi (e_k),\mathrm{{d}}\phi (e_l))\tau (\phi )\rangle \mathrm{{d}}v_g \nonumber \\&= -\int _M\langle \bar{\nabla }_i\big (R^N(\mathrm{{d}}\phi (e_j),\mathrm{{d}}\phi (e_l))R^N(\mathrm{{d}}\phi (e_j),\mathrm{{d}}\phi (e_l))\tau (\phi )\big ),\mathrm{{d}}\phi (e_k)\rangle \omega ^{ki}\mathrm{{d}}v_g \nonumber \\&\quad +\,\frac{1}{2}\int _M\langle \bar{\nabla }^k\big ( R^N(\mathrm{{d}}\phi (e_j),\mathrm{{d}}\phi (e_l))R^N(\mathrm{{d}}\phi (e_j),\mathrm{{d}}\phi (e_l))\tau (\phi )\big ),\mathrm{{d}}\phi (e_k)\rangle \langle \omega ,g\rangle \mathrm{{d}}v_g \nonumber \\&\quad -\,\frac{1}{2}\int _M|R^N(\mathrm{{d}}\phi (e_k),\mathrm{{d}}\phi (e_l))\tau (\phi )|^2\langle \omega ,g\rangle \mathrm{{d}}v_g. \end{aligned}$$

### Proof

Using (3.3) in the first term on the right hand side of (3.2) we obtain

$$\begin{aligned}&\int _M\langle R^N(\mathrm{{d}}\phi (e_k),\mathrm{{d}}\phi (e_l))\frac{\mathrm {d}}{\mathrm {d}t}\big |_{t=0}\tau (\phi ),R^N(\mathrm{{d}}\phi (e_k),\mathrm{{d}}\phi (e_l))\tau (\phi )\rangle \mathrm{{d}}v_g \\&= -\,\int _M\langle R^N(\mathrm{{d}}\phi (e_k),\mathrm{{d}}\phi (e_l))\bar{\nabla }_{i}\mathrm{{d}}\phi (e_j),R^N(\mathrm{{d}}\phi (e_k),\mathrm{{d}}\phi (e_l))\tau (\phi )\rangle \omega ^{ij}\mathrm{{d}}v_g\\&\quad -\,\int _M\langle R^N(\mathrm{{d}}\phi (e_j),\mathrm{{d}}\phi (e_l))\mathrm{{d}}\phi (e_k),R^N(\mathrm{{d}}\phi (e_j),\mathrm{{d}}\phi (e_l))\tau (\phi )\rangle \nabla _i\omega ^{ki}\mathrm{{d}}v_g\\&\quad +\,\frac{1}{2}\int _M\langle R^N(\mathrm{{d}}\phi (e_j),\mathrm{{d}}\phi (e_l))\mathrm{{d}}\phi (e_k),R^N(\mathrm{{d}}\phi (e_j),\mathrm{{d}}\phi (e_l))\tau (\phi )\rangle \nabla ^k{\text {Tr}}\omega \,\mathrm{{d}}v_g. \end{aligned}$$

The first two terms can be manipulated as follows:

$$\begin{aligned} -\int _M\langle&R^N(\mathrm{{d}}\phi (e_k),\mathrm{{d}}\phi (e_l))\bar{\nabla }_{i}\mathrm{{d}}\phi (e_j),R^N(\mathrm{{d}}\phi (e_k),\mathrm{{d}}\phi (e_l))\tau (\phi )\rangle \omega ^{ij}\mathrm{{d}}v_g\\ -\int _M\langle&R^N(\mathrm{{d}}\phi (e_j),\mathrm{{d}}\phi (e_l))\mathrm{{d}}\phi (e_k),R^N(\mathrm{{d}}\phi (e_j),\mathrm{{d}}\phi (e_l))\tau (\phi )\rangle \nabla _i\omega ^{ki}\mathrm{{d}}v_g\\&= \int _M\langle R^N(\mathrm{{d}}\phi (e_k),\mathrm{{d}}\phi (e_l))R^N(\mathrm{{d}}\phi (e_k),\mathrm{{d}}\phi (e_l))\tau (\phi ),\bar{\nabla }_{i}\mathrm{{d}}\phi (e_j)\rangle \omega ^{ij}\mathrm{{d}}v_g\\&\quad +\,\int _M\langle R^N(\mathrm{{d}}\phi (e_j),\mathrm{{d}}\phi (e_l))R^N(\mathrm{{d}}\phi (e_j),\mathrm{{d}}\phi (e_l))\tau (\phi ),\mathrm{{d}}\phi (e_k)\rangle \nabla _i\omega ^{ki}\mathrm{{d}}v_g\\&= -\int _M\langle \bar{\nabla }_i\big (R^N(\mathrm{{d}}\phi (e_j),\mathrm{{d}}\phi (e_l))R^N(\mathrm{{d}}\phi (e_j),\mathrm{{d}}\phi (e_l))\tau (\phi )\big ),\mathrm{{d}}\phi (e_k)\rangle \omega ^{ki}\mathrm{{d}}v_g, \end{aligned}$$

where we first used the symmetries of the Riemann curvature tensor and applied integration by parts in the second step.

Regarding the third term a similar manipulation yields

$$\begin{aligned} \int _M\langle&R^N(\mathrm{{d}}\phi (e_j),\mathrm{{d}}\phi (e_l))\mathrm{{d}}\phi (e_k),R^N(\mathrm{{d}}\phi (e_j),\mathrm{{d}}\phi (e_l))\tau (\phi )\rangle \nabla ^k{\text {Tr}}\omega \,\mathrm{{d}}v_g \\&= \int _M\langle \bar{\nabla }^k\big ( R^N(\mathrm{{d}}\phi (e_j),\mathrm{{d}}\phi (e_l))R^N(\mathrm{{d}}\phi (e_j),\mathrm{{d}}\phi (e_l))\tau (\phi )\big ),\mathrm{{d}}\phi (e_k)\rangle \langle \omega ,g\rangle \mathrm{{d}}v_g \\&\quad -\,\int _M|R^N(\mathrm{{d}}\phi (e_k),\mathrm{{d}}\phi (e_l))\tau (\phi )|^2\langle \omega ,g\rangle \mathrm{{d}}v_g. \end{aligned}$$

The claim then follows from combining the equations. $\square$

We may now give the following

### Proposition 3.4

Let $\phi :M\rightarrow N$ be a smooth map and consider a variation of the metric on $M$ as defined in (3.1). Then the following formula holds

$$\begin{aligned} \frac{\mathrm {d}}{\mathrm {d}t}\big |_{t=0}\frac{1}{2}\int _M&|R^N(\mathrm{{d}}\phi (e_k),\mathrm{{d}}\phi (e_l))\tau (\phi )|^2\mathrm{{d}}v_{g_t}=\int _M\langle {\hat{S}}_4,\omega \rangle \mathrm{{d}}v_g, \end{aligned}$$

where the symmetric tensor ${\hat{S}}_4$ is given by

3.5 $$\begin{aligned} {\hat{S}}_4(X,Y)&:= -\,\langle R^N(\mathrm{{d}}\phi (e_k),\mathrm{{d}}\phi (X))\tau (\phi ),R^N(\mathrm{{d}}\phi (e_k),\mathrm{{d}}\phi (Y))\tau (\phi )\rangle \nonumber \\&\quad -\,\frac{1}{4}|R^N(\mathrm{{d}}\phi (e_k),\mathrm{{d}}\phi (e_l))\tau (\phi )|^2g(X,Y)\nonumber \\&\quad -\,\frac{1}{2}\langle \bar{\nabla }_X\big ( R^N(\mathrm{{d}}\phi (e_k),\mathrm{{d}}\phi (e_l))R^N(\mathrm{{d}}\phi (e_k),\mathrm{{d}}\phi (e_l))\tau (\phi )\big ),\mathrm{{d}}\phi (Y)\rangle \nonumber \\&\quad -\,\frac{1}{2}\langle \bar{\nabla }_Y\big ( R^N(\mathrm{{d}}\phi (e_k),\mathrm{{d}}\phi (e_l))R^N(\mathrm{{d}}\phi (e_k),\mathrm{{d}}\phi (e_l))\tau (\phi )\big ),\mathrm{{d}}\phi (X)\rangle \nonumber \\&\quad +\,\frac{1}{2}\langle \bar{\nabla }^k\big ( R^N(\mathrm{{d}}\phi (e_k),\mathrm{{d}}\phi (e_l))R^N(\mathrm{{d}}\phi (e_k),\mathrm{{d}}\phi (e_l))\tau (\phi )\big ),\mathrm{{d}}\phi (e_k)\rangle \nonumber \\&\quad \times \,g(X,Y). \end{aligned}$$

Here, $X,Y$ are vector fields on $M$.

### Proof

This follows by combining (3.2), (3.4) and symmetrizing the first term on the right hand side of (3.4). $\square$

### Remark 3.5

In terms of the variables $\Omega _0,\Omega _1$ defined in (1.12) we may express (3.5) as follows

3.6 $$\begin{aligned} {\hat{S}}_4(X,Y)&= -\langle \Omega _1(Y),\mathrm{{d}}\phi (X)\rangle +\frac{1}{4}\langle \Omega _0,\tau (\phi )\rangle g(X,Y) \nonumber \\&\quad +\,\frac{1}{2}\langle \bar{\nabla }^k\Omega _0,\mathrm{{d}}\phi (e_k)\rangle g(X,Y) -\frac{1}{2}\langle \bar{\nabla }_X\Omega _0,\mathrm{{d}}\phi (Y)\rangle -\frac{1}{2}\langle \bar{\nabla }_Y\Omega _0,\mathrm{{d}}\phi (X)\rangle . \end{aligned}$$

The trace of (3.5) can easily be computed and yields

$$\begin{aligned} {\text {Tr}}{\hat{S}}_4&= \left( -1-\frac{m}{4}\right) |R^N(\mathrm{{d}}\phi (e_i),\mathrm{{d}}\phi (e_j))\tau (\phi )|^2 \nonumber \\&\quad +\,\left( -1+\frac{m}{2}\right) \langle \bar{\nabla }^k\big ( R^N(\mathrm{{d}}\phi (e_j),\mathrm{{d}}\phi (e_l))R^N(\mathrm{{d}}\phi (e_j),\mathrm{{d}}\phi (e_l))\tau (\phi )\big ),\mathrm{{d}}\phi (e_k)\rangle . \end{aligned}$$

### Remark 3.6

In the case of $M$ being compact we may use integration by parts to deduce

$$\begin{aligned} \int _M{\text {Tr}}{\hat{S}}_4\mathrm{{d}}v=\int _M\left( \frac{m}{4}-2\right) |R^N(\mathrm{{d}}\phi (e_i),\mathrm{{d}}\phi (e_j))\tau (\phi )|^2\mathrm{{d}}v. \end{aligned}$$

This reflects the fact that ES-4-harmonic maps are critical if $\dim M=8$.

Having calculated the variation of $E^\mathrm{{ES}}_4(\phi )$ with respect to the metric on the domain we may now define the stress-energy tensor for ES-4-harmonic maps as follows:

3.7 $$\begin{aligned} S^\mathrm{{ES}}_4(X,Y):=S_4(X,Y)+{\hat{S}}_4(X,Y). \end{aligned}$$

The stress-energy tensor (3.7) satisfies the following conservation law:

### Theorem 3.7

Let $\phi :M\rightarrow N$ be a smooth ES-4-harmonic map, that is a smooth solution of

$$\begin{aligned} \tau _4^\mathrm{{ES}}(\phi )=0, \end{aligned}$$

where $\tau _4^\mathrm{{ES}}(\phi )$ is defined in (1.11). Then the stress-energy tensor defined in (3.7) satisfies

3.8 $$\begin{aligned} {\text {div}}S^\mathrm{{ES}}_{4}=-\langle \tau ^\mathrm{{ES}}_{4}(\phi ),\mathrm{{d}}\phi \rangle . \end{aligned}$$

In particular, the stress-energy tensor is divergence-free whenever $\phi$ is a solution of $\tau _4^\mathrm{{ES}}(\phi )=0$.

### Proof

We choose a local orthonormal basis $e_i,i,\ldots ,m$ around a point $p\in M$ that satisfies $\nabla _ke_r=0$, where $1\le r,k\le m$. Then, we calculate

3.9 $$\begin{aligned} \nabla ^j S^4(e_i,e_j)&= -\,\langle \tau _4(\phi ),\mathrm{{d}}\phi (e_i)\rangle \nonumber \\&= -\,\langle \xi _1,\mathrm{{d}}\phi (e_i)\rangle -\langle \mathrm{{d}}^*\Omega _1,\mathrm{{d}}\phi (e_i)\rangle -\frac{1}{2}\langle \bar{\Delta }\Omega _0,\mathrm{{d}}\phi (e_i)\rangle \nonumber \\&\quad -\,\frac{1}{2}\langle {\text {Tr}}R^N(\mathrm{{d}}\phi (\cdot ),\Omega _0)\mathrm{{d}}\phi (\cdot ),\mathrm{{d}}\phi (e_i)\rangle . \end{aligned}$$

In the first step we made use of (2.2) and in the second step we used that $\phi$ solves $\tau _4^\mathrm{{ES}}(\phi )=0$.

Now, we will show that the right hand side of (3.9) is equal to the negative divergence of ${\hat{S}}^4$. To this end we calculate using (3.6)

3.10 $$\begin{aligned} \nabla ^j{\hat{S}}_4(e_i,e_j)&=-\langle \bar{\nabla }^j\Omega _1(e_j),\mathrm{{d}}\phi (e_i)\rangle - \langle \Omega _1(e_j),\bar{\nabla }_j\mathrm{{d}}\phi (e_i)\rangle \nonumber \\&\quad -\frac{1}{4}\langle \bar{\nabla }_i\Omega _0,\tau (\phi )\rangle + \frac{1}{4}\langle \Omega _0,\bar{\nabla }_i\tau (\phi )\rangle \nonumber \\&\quad +\frac{1}{2}\langle \bar{\Delta }\Omega _0,\mathrm{{d}}\phi (e_i)\rangle + \frac{1}{2}\langle R^N(\mathrm{{d}}\phi (e_i),\mathrm{{d}}\phi (e_k))\Omega _0,\mathrm{{d}}\phi (e_k)\rangle . \end{aligned}$$

Using the definition of $\Omega _1$ given in (1.12) it is easy to derive

3.11 $$\begin{aligned} \langle \Omega _1(e_j),\bar{\nabla }_j\mathrm{{d}}\phi (e_i)\rangle = -\langle R^N(\mathrm{{d}}\phi (e_k),\bar{\nabla }_j\mathrm{{d}}\phi (e_i))\tau (\phi ),R^N(\mathrm{{d}}\phi (e_j),\mathrm{{d}}\phi (e_k))\tau (\phi )\rangle . \end{aligned}$$

Moreover, a direct calculation yields

3.12 $$\begin{aligned} \langle \Omega _0,\bar{\nabla }_i\tau (\phi )\rangle =-\langle R^N(\mathrm{{d}}\phi (e_k),\mathrm{{d}}\phi (e_j))\bar{\nabla }_i\tau (\phi ),R^N(\mathrm{{d}}\phi (e_k),\mathrm{{d}}\phi (e_j))\tau (\phi )\rangle . \end{aligned}$$

In addition, we find by a direct calculation

3.13 $$\begin{aligned} \langle \bar{\nabla }_i\Omega _0,\tau (\phi )\rangle&= 2\langle (\nabla _{\mathrm{{d}}\phi (e_i)}R^N)(\mathrm{{d}}\phi (e_k),\mathrm{{d}}\phi (e_j))R^N(\mathrm{{d}}\phi (e_k),\mathrm{{d}}\phi (e_j))\tau (\phi ),\tau (\phi )\rangle \nonumber \\&\quad -\,4\langle R^N(\bar{\nabla }_i \mathrm{{d}}\phi (e_k),\mathrm{{d}}\phi (e_l))\tau (\phi ),R^N(\mathrm{{d}}\phi (e_k),\mathrm{{d}}\phi (e_l))\tau (\phi )\rangle \nonumber \\&\quad -\,\langle R^N(\mathrm{{d}}\phi (e_k),\mathrm{{d}}\phi (e_l))\bar{\nabla }_i \tau (\phi ),R^N(\mathrm{{d}}\phi (e_k),\mathrm{{d}}\phi (e_l))\tau (\phi )\rangle . \end{aligned}$$

Moreover, we manipulate

$$\begin{aligned} \langle (\nabla&_{\mathrm{{d}}\phi (e_i)}R^N)(\mathrm{{d}}\phi (e_k),\mathrm{{d}}\phi (e_j))R^N(\mathrm{{d}}\phi (e_k),\mathrm{{d}}\phi (e_j))\tau (\phi ),\tau (\phi )\rangle \\&= -\,\langle (\nabla _{\mathrm{{d}}\phi (e_j)}R^N)(\mathrm{{d}}\phi (e_i),\mathrm{{d}}\phi (e_k))R^N(\mathrm{{d}}\phi (e_k),\mathrm{{d}}\phi (e_j))\tau (\phi ),\tau (\phi )\rangle \\&\quad -\,\langle (\nabla _{\mathrm{{d}}\phi (e_k)}R^N)(\mathrm{{d}}\phi (e_j),\mathrm{{d}}\phi (e_i))R^N(\mathrm{{d}}\phi (e_k),\mathrm{{d}}\phi (e_j))\tau (\phi ),\tau (\phi )\rangle \\&= -\,2\langle (\nabla _{\mathrm{{d}}\phi (e_j)}R^N)(\mathrm{{d}}\phi (e_i),\mathrm{{d}}\phi (e_k))R^N(\mathrm{{d}}\phi (e_k),\mathrm{{d}}\phi (e_j))\tau (\phi ),\tau (\phi )\rangle \\&= -\,2\langle (\nabla _{\mathrm{{d}}\phi (e_j)}R^N)(\tau (\phi ),R^N(\mathrm{{d}}\phi (e_k),\mathrm{{d}}\phi (e_j))\tau (\phi ))\mathrm{{d}}\phi (e_k),\mathrm{{d}}\phi (e_i)\rangle , \end{aligned}$$

where we first used the second Bianchi identity and afterwards the symmetries of the Riemannian curvature tensor in the second and third step.

Combining (3.11), (3.12) and (3.13) we get

$$\begin{aligned} -\langle \Omega _1(e_j),\bar{\nabla }_j\mathrm{{d}}\phi (e_i)\rangle -\frac{1}{4}\langle \bar{\nabla }_i\Omega _0,\tau (\phi )\rangle +\frac{1}{4}\langle \Omega _0,\bar{\nabla }_i\tau (\phi )\rangle =\langle \xi _1,\mathrm{{d}}\phi (e_i)\rangle , \end{aligned}$$

and together with (3.10) this completes the proof. $\square$

### Remark 3.8

It was to be expected that the stress-energy tensor associated with the ES-4-energy (1.10) is divergence free. The energy functional (1.10) is invariant under diffeomorphisms on the domain $u:M\rightarrow M$ in the following sense

$$\begin{aligned} E_4^\mathrm{{ES}}(\phi \circ u,u^*g)=E_4^\mathrm{{ES}}(\phi ,g). \end{aligned}$$

This can be explicitly checked with the methods presented in [5, Section 2.3]. Via Noether’s theorem the invariance of the energy functional (1.10) leads to a conserved quantity which is precisely the stress-energy tensor (3.7).

## Proof of Theorem 1.2

In this section, we will prove Theorem 1.2. Our method of proof will be the same as in the proof of Theorem 1.1 but instead of the stress-energy tensor for 4-harmonic maps (1.8) we will now make use of the stress-energy tensor for ES-4-harmonic maps given by (3.7).

As the stress-energy tensor for ES-4-harmonic maps consists of the stress-energy tensor for 4-harmonic maps and an additional piece arising from the curvature term ${\hat{E}}^\mathrm{{ES}}_4(\phi )$, which is given by ${\hat{S}}^4$, we will only have to deal with ${\hat{S}}^4$ as the rest of the calculation will be identical to the one for 4-harmonic maps.

Choosing the same cutoff function as in the proof of Theorem 1.1 and due to the conservation law (3.8) we have

$$\begin{aligned} 0=-\int _{\mathbb {R}^m}\langle x\eta (r),{\text {div}}S^\mathrm{{ES}}_4\rangle \mathrm{{d}}v=\int _{\mathbb {R}^m}\frac{\partial }{\partial x^j}\big (x^i\eta (r)\big )S^\mathrm{{ES}}_4(e_i,e_j)\mathrm{{d}}v. \end{aligned}$$

Inserting the second term from (3.7) into the above equation we find

$$\begin{aligned} \int _{\mathbb {R}^m}&{\hat{S}}_4(e_i,e_j)\delta _{ij}\eta (r)\mathrm{{d}}v \\&= \int _{\mathbb {R}^m}\eta (r)\big (\left( -1-\frac{m}{4}\right) |R^N(\mathrm{{d}}\phi (e_k),\mathrm{{d}}\phi (e_l))\tau (\phi )|^2\mathrm{{d}}v \\&\quad +\,(-1+\frac{m}{2})\langle \bar{\nabla }^k\big ( R^N(\mathrm{{d}}\phi (e_j),\mathrm{{d}}\phi (e_l))R^N(\mathrm{{d}}\phi (e_j),\mathrm{{d}}\phi (e_l))\tau (\phi )\big ),\mathrm{{d}}\phi (e_k)\rangle \big )\mathrm{{d}}v. \end{aligned}$$

Using integration by parts we find

$$\begin{aligned} \int _{\mathbb {R}^m}&\eta (r)\langle \bar{\nabla }^k\big ( R^N(\mathrm{{d}}\phi (e_j),\mathrm{{d}}\phi (e_l))R^N(\mathrm{{d}}\phi (e_j),\mathrm{{d}}\phi (e_l))\tau (\phi )\big ),\mathrm{{d}}\phi (e_k)\rangle \mathrm{{d}}v \\&= -\,\int _{\mathbb {R}^m}\big (\eta (r)\big )_k\langle R^N(\mathrm{{d}}\phi (e_j),\mathrm{{d}}\phi (e_l))R^N(\mathrm{{d}}\phi (e_j),\mathrm{{d}}\phi (e_l))\tau (\phi ),\mathrm{{d}}\phi (e_k)\rangle \mathrm{{d}}v \\&\quad +\,\int _{\mathbb {R}^m}\eta (r)|R^N(\mathrm{{d}}\phi (e_k),\mathrm{{d}}\phi (e_l))\tau (\phi )|^2\mathrm{{d}}v. \end{aligned}$$

Consequently, we obtain

4.1 $$\begin{aligned}&\int _{\mathbb {R}^m}{\hat{S}}_4(e_i,e_j)\delta _{ij}\eta (r)\mathrm{{d}}v \nonumber \\&=(-2+\frac{m}{4})\int _{\mathbb {R}^m}\eta (r)|R^N(\mathrm{{d}}\phi (e_k),\mathrm{{d}}\phi (e_l))\tau (\phi )|^2 \mathrm{{d}}v\nonumber \\&\quad +\,(1-\frac{m}{2})\int _{\mathbb {R}^m}\big (\eta (r)\big )_k\langle R^N(\mathrm{{d}}\phi (e_j),\mathrm{{d}}\phi (e_l))R^N(\mathrm{{d}}\phi (e_j),\mathrm{{d}}\phi (e_l))\tau (\phi ),\mathrm{{d}}\phi (e_k)\rangle \mathrm{{d}}v. \end{aligned}$$

Moreover, we find

$$\begin{aligned} \int _{\mathbb {R}^m}&{\hat{S}}_4(e_i,e_j)\frac{x_i x_j}{r}\eta '(r)\mathrm{{d}}v \\&= -\,\frac{1}{4}\int _{\mathbb {R}^m}\eta '(r)r|R^N(\mathrm{{d}}\phi (e_i),\mathrm{{d}}\phi (e_j))\tau (\phi )|^2\mathrm{{d}}v\\&\quad +\,\frac{1}{2}\int _{\mathbb {R}^m}\eta '(r)r\langle \bar{\nabla }^k\big ( R^N(\mathrm{{d}}\phi (e_j),\mathrm{{d}}\phi (e_l))R^N(\mathrm{{d}}\phi (e_j),\mathrm{{d}}\phi (e_l))\tau (\phi )\big ),\mathrm{{d}}\phi (e_k)\rangle \mathrm{{d}}v\\&\quad -\,\int _{\mathbb {R}^m}\eta '(r)\frac{x_i x_j}{r}\langle R^N(\mathrm{{d}}\phi (e_k),\mathrm{{d}}\phi (e_i))\tau (\phi ),R^N(\mathrm{{d}}\phi (e_k),\mathrm{{d}}\phi (e_j))\tau (\phi )\rangle \mathrm{{d}}v \\&\quad -\,\int _{\mathbb {R}^m}\eta '(r)\frac{x_i x_j}{r}\langle \bar{\nabla }_i\big ( R^N(\mathrm{{d}}\phi (e_k),\mathrm{{d}}\phi (e_l))R^N(\mathrm{{d}}\phi (e_k),\mathrm{{d}}\phi (e_l))\tau (\phi )\big ),\mathrm{{d}}\phi (e_j)\rangle \mathrm{{d}}v. \end{aligned}$$

In addition, it is straightforward to manipulate

$$\begin{aligned} \int _{\mathbb {R}^m}&\eta '(r)r\langle \bar{\nabla }^k\big ( R^N(\mathrm{{d}}\phi (e_j),\mathrm{{d}}\phi (e_l))R^N(\mathrm{{d}}\phi (e_j),\mathrm{{d}}\phi (e_l))\tau (\phi )\big ),\mathrm{{d}}\phi (e_k)\rangle \mathrm{{d}}v\\&= -\,\int _{\mathbb {R}^m}\big (\eta '(r)r\big )_k\langle R^N(\mathrm{{d}}\phi (e_j),\mathrm{{d}}\phi (e_l))R^N(\mathrm{{d}}\phi (e_j),\mathrm{{d}}\phi (e_l))\tau (\phi ),\mathrm{{d}}\phi (e_k)\rangle \mathrm{{d}}v \\&\quad +\,\int _{\mathbb {R}^m}\eta '(r)r|R^N(\mathrm{{d}}\phi (e_i),\mathrm{{d}}\phi (e_j))\tau (\phi )|^2 \mathrm{{d}}v \end{aligned}$$

and also

$$\begin{aligned} \int _{\mathbb {R}^m}&\eta '(r)\frac{x_i x_j}{r}\langle \bar{\nabla }_i\big ( R^N(\mathrm{{d}}\phi (e_k),\mathrm{{d}}\phi (e_l))R^N(\mathrm{{d}}\phi (e_k),\mathrm{{d}}\phi (e_l))\tau (\phi )\big ),\mathrm{{d}}\phi (e_j)\rangle \mathrm{{d}}v \\&= -\,\int _{\mathbb {R}^m}\left( \eta '(r)\frac{x_i x_j}{r}\right) _i\langle R^N(\mathrm{{d}}\phi (e_k),\mathrm{{d}}\phi (e_l))R^N(\mathrm{{d}}\phi (e_k),\mathrm{{d}}\phi (e_l))\tau (\phi ),\mathrm{{d}}\phi (e_j)\rangle \mathrm{{d}}v\\&\quad +\,\int _{\mathbb {R}^m}\eta '(r)\frac{x_i x_j}{r}\langle R^N(\mathrm{{d}}\phi (e_k),\mathrm{{d}}\phi (e_l))\bar{\nabla }_i\mathrm{{d}}\phi (e_j),R^N(\mathrm{{d}}\phi (e_k),\mathrm{{d}}\phi (e_l))\tau (\phi )\rangle \mathrm{{d}}v. \end{aligned}$$

Consequently, we get

4.2 $$\begin{aligned}&\int _{\mathbb {R}^m}{\hat{S}}_4(e_i,e_j)\frac{x_i x_j}{r}\eta '(r)\mathrm{{d}}v\nonumber \\&\quad = \frac{1}{4}\int _{\mathbb {R}^m}\eta '(r)r|R^N(\mathrm{{d}}\phi (e_i),\mathrm{{d}}\phi (e_j))\tau (\phi )|^2\mathrm{{d}}v\nonumber \\&\quad \quad -\,\frac{1}{2}\int _{\mathbb {R}^m}\big (\eta '(r)r\big )_k\langle R^N(\mathrm{{d}}\phi (e_j),\mathrm{{d}}\phi (e_l))R^N(\mathrm{{d}}\phi (e_j),\mathrm{{d}}\phi (e_l))\tau (\phi ),\mathrm{{d}}\phi (e_k)\rangle \mathrm{{d}}v \nonumber \\&\quad \quad -\,\int _{\mathbb {R}^m}\eta '(r)\frac{x_i x_j}{r}\langle R^N(\mathrm{{d}}\phi (e_k),\mathrm{{d}}\phi (e_i))\tau (\phi ),R^N(\mathrm{{d}}\phi (e_k),\mathrm{{d}}\phi (e_j))\tau (\phi )\rangle \mathrm{{d}}v \nonumber \\&\quad \quad +\,\int _{\mathbb {R}^m}\left( \eta '(r)\frac{x_i x_j}{r}\right) _i\langle R^N(\mathrm{{d}}\phi (e_k),\mathrm{{d}}\phi (e_l))R^N(\mathrm{{d}}\phi (e_k),\mathrm{{d}}\phi (e_l))\tau (\phi ),\mathrm{{d}}\phi (e_j)\rangle \mathrm{{d}}v\nonumber \\&\quad \quad -\,\int _{\mathbb {R}^m}\eta '(r)\frac{x_i x_j}{r}\langle R^N(\mathrm{{d}}\phi (e_k),\mathrm{{d}}\phi (e_l))\bar{\nabla }_i\mathrm{{d}}\phi (e_k),R^N(\mathrm{{d}}\phi (e_j),\mathrm{{d}}\phi (e_l))\tau (\phi )\rangle \mathrm{{d}}v. \end{aligned}$$

To estimate the terms in (4.1) and (4.2) we use Young’s inequality in the following form

$$\begin{aligned} \langle R^N(\mathrm{{d}}\phi (e_j),\mathrm{{d}}\phi (e_l))R^N(\mathrm{{d}}\phi (e_j),\mathrm{{d}}\phi (e_l))\tau (\phi ),\mathrm{{d}}\phi (e_k)\rangle&\le C|\mathrm{{d}}\phi |^5|\bar{\nabla }\mathrm{{d}}\phi | \\&\le C\left( |\mathrm{{d}}\phi |^6+|\mathrm{{d}}\phi |^4|\bar{\nabla }\mathrm{{d}}\phi |^2\right) . \end{aligned}$$

Together with the estimates on the cutoff function (2.6), (2.7) and the estimates obtained in the proof of Theorem 1.1, which are (2.3), (2.4) and (2.5), we get the following inequality

$$\begin{aligned} \int _{\mathbb {R}^m}&\eta (r)(|\bar{\Delta }\tau (\phi )|^2\mathrm{{d}}v+\frac{1}{2}|R^N(\mathrm{{d}}\phi (e_k),\mathrm{{d}}\phi (e_l))\tau (\phi )|^2)\mathrm{{d}}v \\&\le \frac{C}{|8-m|}\big (\frac{1}{R}+\frac{1}{R^2}+\frac{1}{R^3}\big ) \int _{\mathbb {R}^m}(|\mathrm{{d}}\phi |^2+|\bar{\nabla }\mathrm{{d}}\phi |^2+|\bar{\nabla }^2\mathrm{{d}}\phi |^2+|\bar{\nabla }^3\mathrm{{d}}\phi |^2)\mathrm{{d}}v \\&\quad +\,\frac{C}{|8-m|}\frac{1}{R} \int _{\mathbb {R}^m}(|\bar{\nabla }\mathrm{{d}}\phi |^2|\mathrm{{d}}\phi |^4+|\mathrm{{d}}\phi |^6)\mathrm{{d}}v\\&\quad +\,\frac{C}{|8-m|}\int _{B_{2R}\setminus B_R}(|\bar{\Delta }\tau (\phi )|^2+|\mathrm{{d}}\phi |^4|\tau (\phi )|^2)\mathrm{{d}}v. \end{aligned}$$

As long as $\dim M\ne 8$ we can take the limit $R\rightarrow \infty$ and using the finiteness assumption (1.14) the calculation from above yields that

$$\begin{aligned} \bar{\Delta }\tau (\phi )=0,\qquad R^N(\mathrm{{d}}\phi (e_i),\mathrm{{d}}\phi (e_j))\tau (\phi )=0. \end{aligned}$$

The claim now follows by the same arguments given at the end of the proof of Theorem 1.1.
